# Effect of Pack Chromizing on Microstructure and Tribological Properties of GCr15 Bearing Steel

**DOI:** 10.3390/molecules30183690

**Published:** 2025-09-10

**Authors:** Dejun Yan, Chunbei Wei, Peng Tang, Shuqi Huang, Songsheng Lin, Qian Shi, Xiaodong Hong

**Affiliations:** 1 Guangdong Provincial Key Laboratory of Advanced Welding Technology for Ships, School of Materials and Energy, Foshan University, Foshan 528000, China; yandejun_2003@163.com; 2 State Key Laboratory of Special Materials Surface Engineering, National Engineering Laboratory of Modern Materials Surface Engineering Technology, Guangdong Provincial Key Laboratory of Modern Surface Engineering Technology, Institute of New Materials, Guangdong Academy of Sciences, Guangzhou 510651, China; tangpeng@gdinm.com (P.T.); huangshuqi@gdinm.com (S.H.); linsongsheng@gdinm.com (S.L.); shiqian@gdinm.com (Q.S.)

**Keywords:** pack chromizing, chromizing time, hardness, indentation, tribological properties

## Abstract

Chromizing layers are widely employed in industrial applications due to their superior wear resistance and corrosion resistance. In this study, GCr15 bearing steel was chromized by a solid powder pack chromizing method, and the influence of chromizing time on the microstructure and mechanical properties of the chromized layers was systematically investigated. The results reveal the presence of fine pores dispersed both on the surface and at the chromized layers/substrate interface. The concentration of the Cr and Fe elements displays a gradient distribution throughout the layers. The chromized layers are primarily composed of (Cr,Fe)_23_C_6_ and (Cr,Fe)_7_C_3_ phases. With an increase in the chromizing time, the thickness and hardness of the chromized layers are gradually increased. A large number of radial and circumferential cracks are observed both within and around the indentation regions, accompanied by spalling at the edge. The brittleness of the chromized layer is increased, and the spalling phenomenon becomes more pronounced with prolonged chromizing time. The chromizing treatment significantly improves the tribological performance of GCr15 steel, reducing its wear rate to approximately one fifth of that of the untreated substrate.

## 1. Introduction

GCr15 steel, one of the most representative high-carbon and low-alloy steel types, accounts for 80% of bearing steel production due to its low cost, excellent wear resistance, high strength and hardness. It has been widely used in critical mechanical components such as bearing rings, ball screws, and steel balls across marine ships and the aviation, railway and automotive industries [1,2,3]. With the manufacturing techniques and processing methods improving, the bearing life has been enhanced more than 400 times compared with 80 years ago [4]. However, under increasingly demanding service conditions involving heavy loads, elevated temperatures and high-speed cyclic stresses, bearing components remain vulnerable to premature failure through wear, fatigue and corrosion. Especially in the marine environment, GCr15 is subjected to severe electrochemical corrosion and wear, which significantly compromises its applicability in such settings. Therefore, it is of great importance to improve the wear resistance, corrosion resistance and high fatigue strength of the GCr15 bearing steel.

To enhance surface properties, various approaches have been developed, including surface coatings [5,6], surface alloys [7,8], surface texturing [9], strengthen grinding [1,10], and heat treatment [4,11]. Chromium and its ceramic coatings made from carbides or nitrides are particularly promising due to their high hardness, wear resistance, electrochemical corrosion resistance and oxidation stability, even under harsh environmental conditions. Our previous research (not given in this paper) and a large number of studies show that the chromium and its ceramic coatings have excellent electrochemical corrosion resistance [12,13,14]. However, its wear resistance is highly dependent on the coating structure and fabrication process. Thus, an in-depth investigation into the factors influencing the wear resistance of the chromium-based coatings is of significant importance.

Chromium-based coatings can be produced by various techniques, such as physical vapor deposition (PVD) [15], chemical vapor deposition (CVD) [16], thermal spraying (TS) [17] and thermos-reactive diffusion (TRD) [18]. Among these, the TRD method offers distinct advantages for great adhesion strength of coatings to the substrate, the formation of uniform, continuous and dense coating with better hardness, and wear resistance, as well as a low friction coefficient. Consequently, TRD has been widely used to produce hard carbide and nitride coatings with excellent physical and mechanical properties on tool steels, such as forging, extrusion, plastics, glass working, powder metallurgy and cutting tools molds working [19,20,21].

Substantially, the main factors affecting the microstructure and properties of TRD coatings are as follows: process time, treatment temperature, chemical potential of the carbon interstitial element, and chemical composition of the substrate. Considering the fact that the diffusion of carbon atoms in the austenitic substrate is much faster and easier than the diffusion of other elements in the carbide coating layer, the growth rate and pattern of the chromium carbide layer mainly rest on the outward diffusion of C atoms in the steel substrate. Therefore, the carbon content of the steel substrate significantly affects the formation mechanism, microstructure and properties of the chromized layers [20,22,23]. According to this, chromization is usually divided into two categories: soft chromization and hard chromization. The former is employed on the alloys with less than 0.1 wt.% carbon to improve their corrosion and high-temperature oxidation properties. Layers of chromium-rich phases were observed, which contributes to long-term stability of corrosion resistance [24,25,26], whereas a hard chromization process is applied to the alloys with carbon contents exceeding 0.3 wt.% to achieve a high-hardness surface layer. This process generates hard chromium carbide phases, resulting in superior mechanical properties, including increased hardness, strong adhesion and enhanced wear resistance [21,22,23,27]. Lee J.W. et al. [28] studied the influence of carbon contents on the mechanical properties of steels after they were treated by the hard chromization process. It indicated that the thickness of the chromized layer obeys the parabolic rate law and increases with the chromizing time and carbon contents of the substrate. The primary phase on the chromized surface is (Cr,Fe)_2_N_1−x_ with small amount of (Cr,Fe)_23_C_6_. The thickness of the surface nitrogen-rich layer, i.e., the (Cr,Fe)_2_N_1−x_ phase, increases with carbon concentration and chromizing time; this can contribute to the higher critical loads and sufficient adhesion properties in the high C content substrate.

GCr15 bearing steel is a high-carbon steel with a carbon content as high as 1 wt.%. Several studies have investigated chromizing treatment for GCr15 substrate. Tan C et al. [29] fabricated chromized coatings on GCr15 bearing steel with a gas-phase chromizing technique. The coatings exhibited superior wear resistance due to their high hardness (16.6 GPa) and interfacial adhesion (~50 N). The wear rate of the chromized layer decreased dramatically from 2.03×10^−5^ mm^3^/(N.m) to 1.8×10^−6^ mm^3^/(N.m) as the load increased from 10 N to 25 N, which attributes to the formation of chromium oxide tribolayer via shear-induced oxidation. Wang H. et al. [30] prepared chromized coatings on GCr15 steel surface with a solid powder pack chromizing method (TRD) and found a dual-layer chromized structure: a porous outer layer composed mainly of (Cr,Fe)_23_C_6_ phase and a dense inner layer dominated by (Cr,Fe)_7_C_3_ phase. The high C content in GCr15 substrate promotes the formation of the porous (Cr,Fe)_23_C_6_ layer and reduces the diffusion rate of Cr. Chromizing treatment significantly improves the hardness of the GCr15 substrate, which exceeds 1700 HV. Zong X. et al. [31] fabricated chromium carbide coatings (Cr_7_C_3_ and (Cr,Fe)_7_C_3_) on the surface of AISI 52100 steel (equivalent to GCr15) using the TRD. They found that the hardness of the surface increases to 1730–1920 HV and the adhesion-strength quality of the coating is correlated to HF2~HF3 according to the VDI 3198 standards [32]. The chromium carbide coating can significantly improve the wear resistance of the substrate with the friction coefficient decreased from 0.46 to 0.37 and wear-weight loss decreased by 89.3%.

Despite the potential advantages mentioned above, the application of TRD technology in GCr15 steel is not extensive compared with other deposition methods. Furthermore, limited research has focused on the adhesion strength and tribological properties, particularly regarding the wear-failure mechanism of chromized layers on bearing steels. Therefore, a comprehensive investigation of TRD’s detailed effects on the microstructure, adhesion, and tribological properties of GCr15 steel is warranted. Among the TRD methods, solid powder pack chromizing is a relatively mature process for preparing the chromized layer with a simple method and low cost [33]. In this study, solid powder pack chromizing treatment was applied to GCr15 bearing steel to improve its wear resistance. The adhesion strength and the tribological behavior of the chromized layers were systematically investigated to comprehend the relationships between composition, microstructure and wear properties.

## 2. Results and Discussion

### 2.1. Microstructure and Compositions

**Figure 1:**
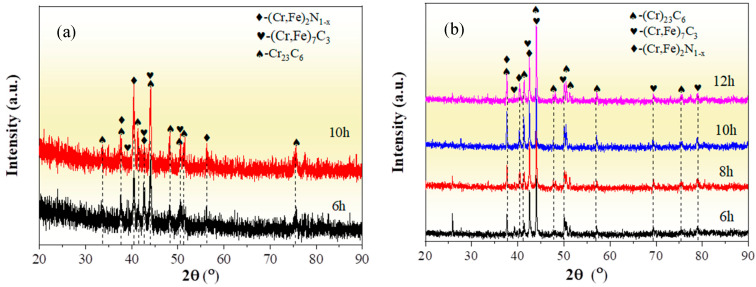
XRD patterns of the chromized layers with different chromizing time (**a**) grazing incidence; (**b**) normal incidence.

Figure 1 depicts the XRD patterns of the chromized layers treated under different chromizing time by both grazing-incidence XRD (GIXRD) and conventional XRD measurements. The chromized layer primarily consists of Cr_23_C_6_ (PDF#35-0783) and (Cr,Fe)_7_C_3_ (PDF# 05-0720) phases, accompanied by a minor (Cr,Fe)_2_N_1−x_ (PDF#19-0330) phase. The enhanced intensity of (Cr,Fe)_2_N_1−x_ diffraction peaks in GIXRD indicates that its preferential disperses on the surface of the chromized layer. The nitrogen in the chromized layer is primarily derived from the decomposition of NH_4_Cl in the chromizing agent. The decomposed nitrogen atoms react with the surface-adsorbed Cr to form (Cr,Fe)_2_N_1−x_.

The formation process of the chromized layer primarily involves the inward diffusion of Cr, N atoms from the chromizing agent and the outward diffusion of substrate elements such as Fe and C. In the initial stage, the following reactions (as shown in Table 1 Equations (1)–(8)) occur in the chromizing agent at high temperatures [34,35].

It is known that the growth of the chromized layer involves thermodynamics and kinetics issues. At a low temperature (<1000 °C), the resistance encountered during the coating growth is high and the kinetics dominate the growth process of coating. The Cr atoms/ions spill from the packed powders and diffuse into the substrate at a slow rate [36]. Cr atoms diffuse inwards and C atoms in the substrate diffuse outwards, meeting at the interface and reacting to form the chromium carbide phase. Theoretically, there would be many different types of chromium carbides including Cr_23_C_6_, Cr_7_C_3_ and Cr_3_C_2_, formed by the reaction. The following equations (as shown in Table 1 Equations (9)–(11)) show the reaction and the associated Gibbs free energy [21].

Previous studies indicate that the Gibbs free energy of Cr_23_C_6_, Cr_7_C_3_ and Cr_3_C_2_ remains nearly constant within a certain temperature range. According to the thermodynamic theory, it can be concluded that the formation order of the three kinds of chromium carbides should by Cr_23_C_6_ first, then Cr_7_C_3_ and lastly Cr_3_C_2_ [37]. Thus, when the ratio between Cr and C is large enough, Cr_23_C_6_ phase can easily form [38]. The phase structure of the chromized layer is also closely related to the C content in the substrate. For high-carbon steel, the active chromium atoms first react with the carbon atoms on the surface of the steel substrate to form Cr_23_C_6_. After the formation of the Cr_23_C_6_ layer on the surface, surface-adsorbed Cr atoms penetrate through the carbide layer to reach the substrate, which increases the inward diffusion resistance of Cr atoms, while the Cr atoms in the Cr_23_C_6_ phase near the substrate interface constantly diffuse inward. This results in a continuous decrease in Cr content in the Cr_23_C_6_ phase at the interface, coupled with the continuous outward diffusion of C atoms from the substrate, which promote the phase transformation from Cr_23_C_6_ to Cr_7_C_3_ near the interface [27]. Additionally, the outward diffusion of Fe from the substrate to replace part of the Cr atoms is more conducive to thermodynamic stability [39]. So, the (Cr,Fe)_23_C_6_ phase and the (Cr,Fe)_7_C_3_ phase are formed in the chromized layer. In Figure 1b, with the increase in chromizing time, the diffraction peak intensity enhances, indicating that the crystal structure of the chromized layer phase is strengthened, which may be attributed to the grain growth caused by the prolonged chromizing time.

According to the Scherrer equation,

(12) $$D_{hkl}=\frac{K\lambda }{\beta_{hkl}\cos\theta }$$

In the formula, *K* is a constant, β is a half-height width of the diffraction peak, *θ* is an angle corresponding to the diffraction peak, and *λ* is a wavelength of the x-ray. Bringing the angle of a single diffraction peak into Equation (12), the grain thickness of the corresponding crystal plane normal is obtained. After taking multiple angles into the calculation, the grain size *D* can be obtained by averaging. The calculation results of grain size under different chromizing time are summarized in Table 2. It can be observed that as the chromizing time increases, the grain size of the Cr_23_C_6_ phase increases gradually, while the grain size of the (Cr,Fe)_7_C_3_ phase does not exhibit a significant change. In summary, the grain size of the chromized layer gradually coarsens with extended chromizing time.

**Figure 2:**
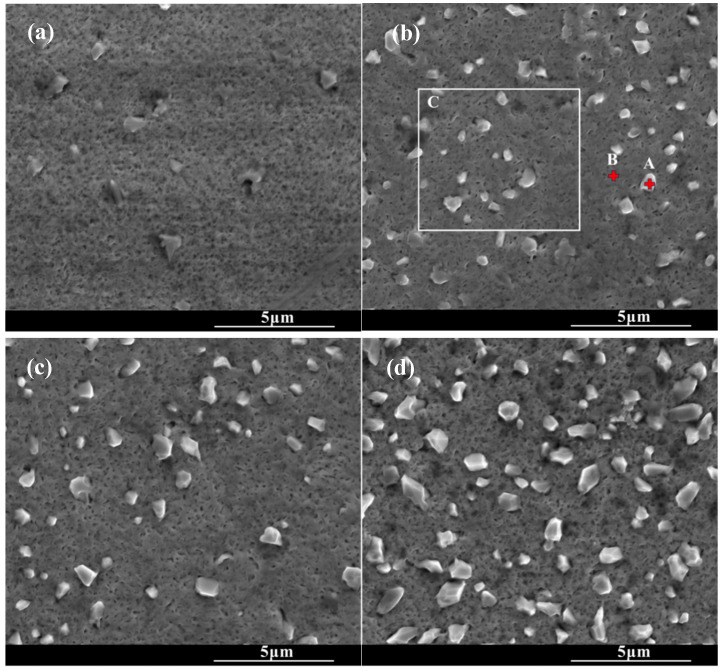
SEM surface morphology of the chromized layers under different chromizing time (**a**) CT6; (**b**) CT8; (**c**) CT10; (**d**) CT12.

Figure 2 shows the surface morphologies of the chromized layers under the different chromizing time. The surface of the chromized layer exhibits relatively smooth topography, with scattered protruding rhombic grains embedded on the surface. As the chromizing time increases, the number and size of rhombic grains gradually increase. A large number of fine pores are distributed on the chromized layer surface. The formation of these pores may be attributed to the migration of vacancies in the subsurface of substrate, inward diffusion of Cr atoms into the substrate and decomposition of gas atoms from the NH_4_Cl activator in the pack mixture [22,40]. EDS analysis was performed on different regions of the chromized surface, as shown in Table 3. It can be found that the protruding rhombic grains are primarily enriched with Cr and N, while the remaining areas consist mainly of Cr and C. This indicates that the chromized layer predominantly comprises chromium carbide phases, while the scattered rhombic grains represent nitrogen-rich compounds. The presence of a small number of nitrogen-rich compounds in the chromized layer is related to active nitrogen atoms decomposed from the activator NH_4_Cl in the chromizing agent that produces N_2_ and combines with active Cr atoms to form Cr–N compounds [34]. This is consistent with the result of XRD.

**Figure 3:**
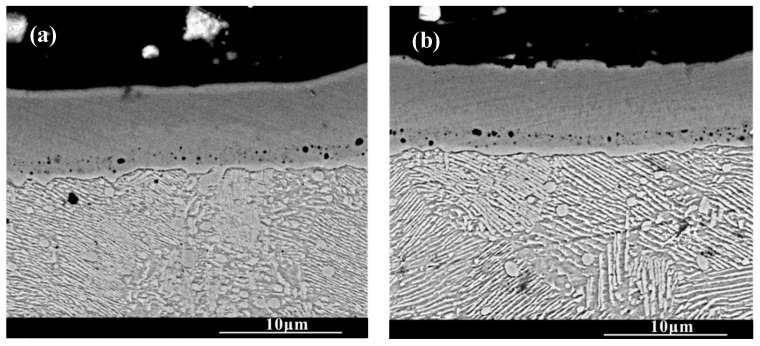

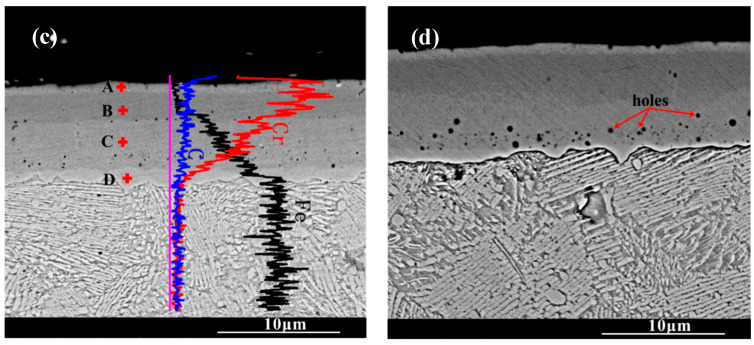
SEM cross-section morphology of the chromized layers with different chromizing time (**a**) CT6; (**b**) CT8; (**c**) CT10; (**d**) CT12.

Figure 3 shows the cross-section morphology of the chromized layer under different chromizing times. The microstructure exhibits distinct light–dark contrast, enabling clear differentiation into four distinct layers labeled A, B, C and D (as shown in Figure 3c). According to the EDS results shown in Table 4, the Cr content in the chromized layer gradually decreases and the Fe content gradually increases from the surface to the substrate, forming a gradual composition transition structure. The C content is higher than that of the substrate throughout the chromized layer due to the outward diffusion from the substrate. It is known that the diffusion coefficient of C atoms is three or four orders of magnitude greater than that of Cr atoms at high temperature. In the early stage of the chromizing process, Cr atoms first deposit on the surface and then diffuse inward; its penetration inside is stopped by a counter-diffusion of carbon as a result of the vigorous chemical affinity of these elements and formation of a carbide layer [39]. No N element is detected inside the chromized layer. A small amount of nitrogen atoms from the decomposition of NH_4_Cl in the agent combine with Cr on the surface to form the chromium nitride phase sporadically dispersed on the surface.

A certain number of pores distribute at the interface of the chromized layer/steel substrate shown in Figure 3. According to Figure 2 and Figure 3, two types of pores are observed in the chromized layer: surface pores and interfacial pores. Previous studies have shown that the formation of pores on the surface of the chromized layer is caused by the mismatch diffusion of Cr atoms and Fe atoms in the chromized layer and the aggregation of vacancy migration at grain boundaries, as well as gas decomposition from the activator (NH_4_Cl in the pack), etc. [30,40,41]. During the chromizing process, Cr atoms diffuse inwards and C atoms in the substrate diffuse outwards, meeting at the interface and reacting to form the chromium carbide phase. Once this carbide layer develops, surface-adsorbed Cr atoms must penetrate it to reach the substrate, significantly hindering inward diffusion and consequently limiting chromized layer growth. The initially formed chromium carbide phase (such as Cr_23_C_6_) can also serve as a Cr source for further inward diffusion. If the vacancies generated by the inward diffusion of Cr atoms cannot be timely, compensated by the inward diffusion of surface Cr atoms from external medium, a large number of vacancies will migrate and coalesce, leading to the formation of pores on the surface [22]. Wang H.F. et al. [30] observed that the formation of pores on the surface was caused by the differential Cr diffusion rates between grain boundaries and intragranular regions in the Cr-rich phase layer. The lower boundaries of most pores are connected to grain boundaries, while the upper boundaries of pores are located within the grains. The diffusion rate of atoms along the grain boundary is much higher than that through intragranular region, resulting in the formation of a large number of vacancies. Vacancies are constantly accumulating, and finally pores are formed. Similarly, the mechanism of pore formation and growth at the chromized layer/steel substrate interface is also the result of this inequality of atom diffusion. Additionally, the stress and relaxation, which are caused by the different specific volumes and coefficients of linear thermal expansion of the carbide layer and the steel substrate and which is accompanied by vacancy coagulation at the interface boundary, may also be causes of porosity at the interface of carbide-layer/steel [39]. Such interfacial porosity may reduce the bonding strength of the chromized layer to the substrate [42].

**Figure 4:**
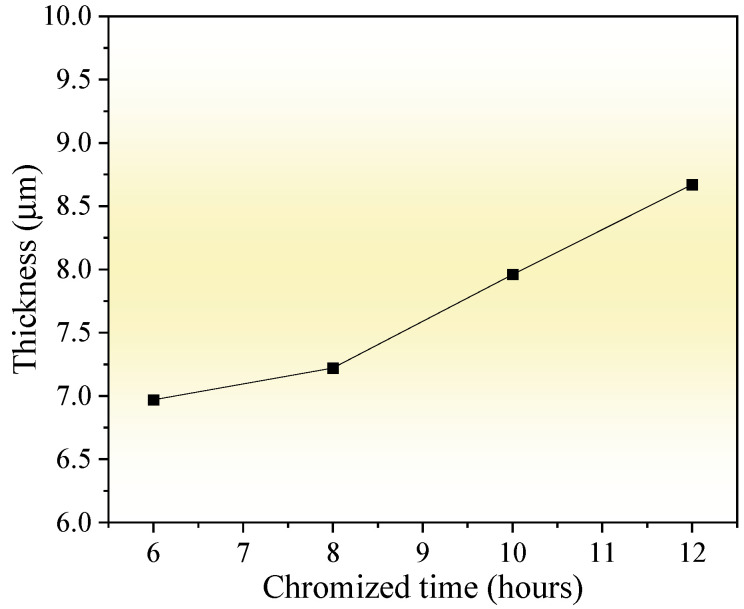
Influence of chromizing time on thickness of the chromized layers.

Figure 4 shows the thickness variation in the chromized layer under different chromizing times. As the chromizing time increases from 6 h to 12 h, the layer thickness exhibits a gradual growth from 6.97 μm to 8.67 μm. Previous research has shown that C content in the substrate significantly influences the thickness of the chromized layer. Liu T. et al. [22] proposed that the carbon content in the substrate of 0.2 wt.% is a cut-off point, which has an apparent influence on the structure and growth mechanism of the chromized layer. When the C content is ≤0.2 wt.%, the thickness of the chromized layer is significantly thicker, although the thickness of the permeable layer decreased slightly with the increase in C content in the substrate (ranging from 51.19 μm to 31.39 μm). In contrast, for substrates with higher carbon content (>0.2 wt.%), the chromized layer remains relatively thin (13.04 μm to 15.60 μm for C content of 0.3~0.45 wt.%) and shows only a slight increase in thickness with further elevation of C content. However, Lee J.W. et al. [28] indicated that the thickness of the chromized layer obeys the parabolic rate law: X = K✓t and increases with chromizing time and carbon contents in the substrate. The high carbon content (up to 1 wt.%) in GCr15 steel substrate promotes the rapid formation of the chromium carbide layer during the chromizing process. After the chromium carbide layer is formed due to the vigorous chemical affinity on the surface, Cr atoms adsorbed on the surface must pass through the carbides layer to reach the substrate, which increases the difficulty of inward diffusion, hindering the inward diffusion of Cr atoms, and thus limiting the growth rate of the chromized layer. As a result, the thickness of the chromized layer increases slightly with an increase in the chromizing time. Since the diffusion rate of C atoms at a high temperature is much higher than that of Cr, the outward diffusion of C atoms has become the main reason for the growth of the chromized layer. As indicated during chromized plating, about 70% of the thickness of the carbide layer is formed due to its outward growth, and only 30% due to inward growth into the depth of specimen [39].

### 2.2. Microhardness

**Figure 5:**
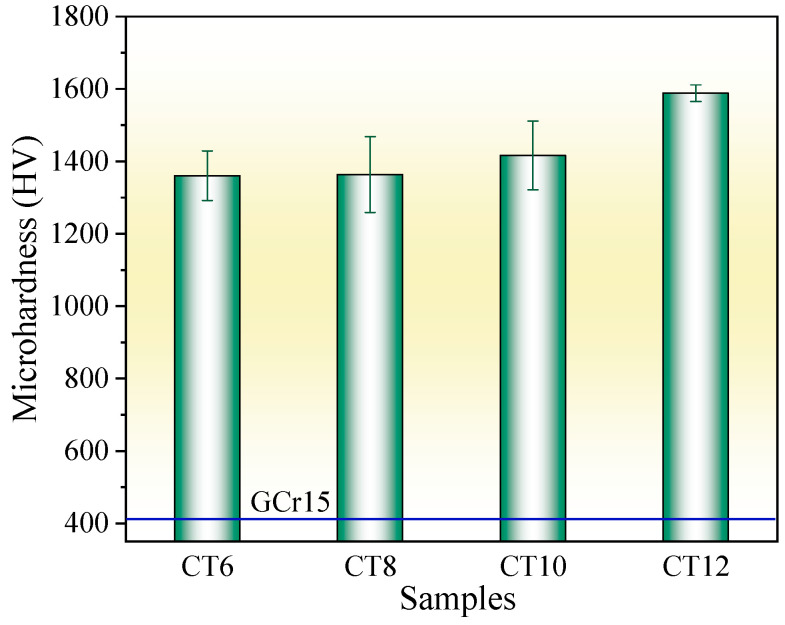
Microhardness of the chromized layers under different chromizing time.

Figure 5 shows the surface microhardness of the chromized layer under different chromizing times. With the increase in chromizing time, the surface hardness of the chromized layer increases, varying from 1360.6 HV to 1588.2 HV. Previous studies indicate that the microhardness of the (Cr,Fe)_23_C_6_ phase is 1200 HV–1400 HV, 2000 HV–2500 HV for (Cr,Fe)_7_C_3_ phase, and 18GPa for (Cr,Fe)_2_N_1−x_ phase [28,40]. As indicated by the XRD results, the chromized layer is mainly composed of (Cr,Fe)_23_C_6_ and (Cr,Fe)_7_C_3_, meaning its overall hardness reflects a composite of these two phases. With the increase in chromizing time, the thickness of the chromized layer increases, while its microstructure and phase composition change slightly. Therefore, the increment of hardness within the chromizing time should be primarily attributed to the improvement of layer thickness. Compared with that of the GCr15 bearing steel substrate (412 HV), the chromizing treatment improves the surface hardness by more than three times.

### 2.3. Indentation

**Figure 6:**
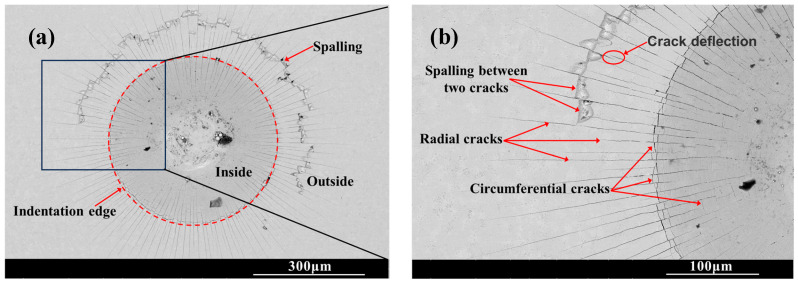

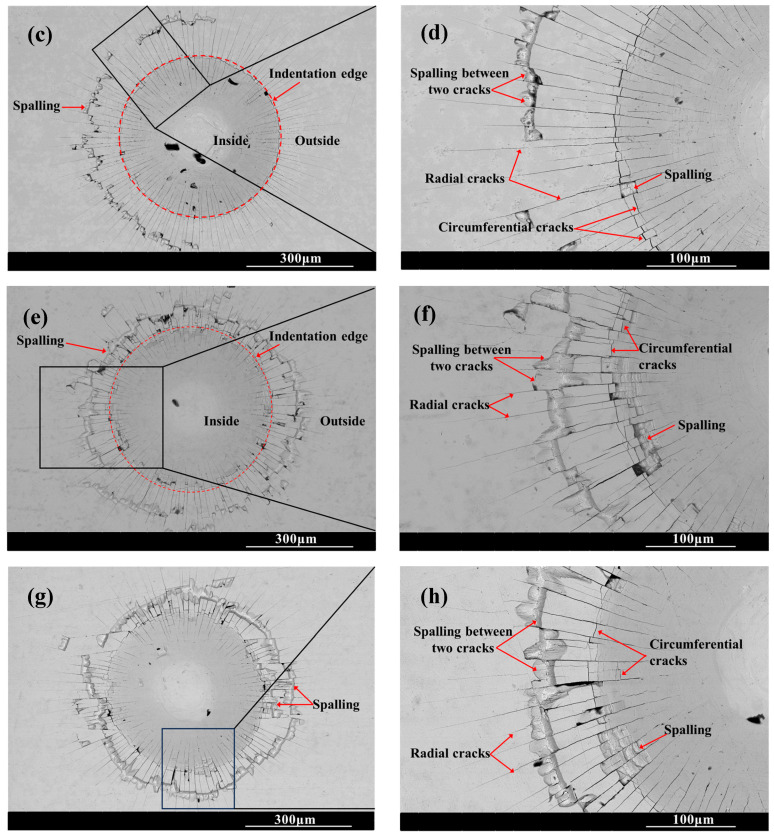
SEM indention morphology of the chromized layers (**a**,**b**) CT6; (**c**,**d**) CT8; (**e**,**f**) CT10; (**g**,**h**) CT12.

Figure 6 shows the SEM morphology of the indentation on the surface of the chromized layer. Numerous cracks exist within and around the indentation, where radial and circumferential cracks are observed inside the indentation zone, while radial cracks dominate the surrounding area. Radial cracks typically initiate inside the indentation, then propagate outward in a radial pattern through the indentation edge. Some radial cracks deflect and then continue to extend outward. Within the indentation zone, circumferential cracks appear to be discontinuous, terminating at radial cracks. That is, numerous discontinuous circumferential cracks exist between radial cracks, with coarser ones appearing at the indentation edge. The presence of circumferential cracks reflects fracture behavior in the material, indicating that the chromized layer inside the indentation has undergone fracturing, with the most severe fracture occurring and leading to the spalling around the indentation edge. Spalling tends to occur in enclosed regions formed by radial and circumferential cracks. The spalling regions surrounding the indention observed for CT6 and CT8 are relatively small, with minimal spalling at the indention edge. In contrast, CT10 and CT12 exhibited obviously larger spalling areas. The spalling zones surrounding the indentations are nearly completely interconnected, forming a continuous spalling circle. Furthermore, spalling at the indentation edge obviously increases and gradually propagates outward, merging with the outer spalling circle to form extensive spalling regions. This indicates that the brittleness of the chromized layer increases with prolonged chromizing time. Burnett P.J. et al. [43] revealed that the ratio of *E_f_*/*E_s_* (where *E_f_* and *E_s_* are the Young’s modulus of coatings and substrate, respectively) significantly influences the indentation-cracking behavior of the coating, primarily depending on the deformation mode of the coating/substrate system. When the elastic modulus of the coating is close to that of the substrate, the coating deformation under stress is obstructed by the substrate, leading to cracking in the coating and typically in radial patterns. If a coating with a higher elastic modulus is deposited onto a relatively softer substrate, the high-hardness coating usually has higher yield stress, and the soft substrate tends to yield first under stress. In this case, the deformation of coating will be affected by the deformation of the substrate; that is, the plastic deformation zone of the substrate beneath the coating will increase the curvature of the coating, thus increasing the cracking displacement and generating frame-like cracks (circumferential cracks). Simulation results confirm that the material beneath the indenter undergoes plastic deformation under compressive stress during the indentation process, being extruded and accumulating/expanding around the indentation. The maximum radial tensile stress exists outside the indentation edge, which is the main reason for the circumferential cracking of the coating. As indentation depth increases, the maximum circumferential tensile stress increases and its position shifts toward the indentation edge [44,45].

The chromized layer forms a continuous metallurgical bond with the steel substrate, ensuring excellent bonding strength between them and preventing premature delamination during loading. During stress loading, the chromized layer bears most of the stress due to its high hardness. However, the grain boundaries and defects within the chromized layer are prone to becoming stress concentration points, where cracks first initiate and propagate. As shown in Figure 2 and Figure 3, there are many pores on the surface and at the interface of the chromized layer/steel substrate. These pores may accelerate the propagation of cracks and lead to the spalling of layers. Furthermore, with the increase in chromizing time, the grains of the chromized layer are coarsened, causing the increment of the brittleness of the chromized layer and resulting in serious spalling around the indentation.

### 2.4. Tribological Properties

**Figure 7:**
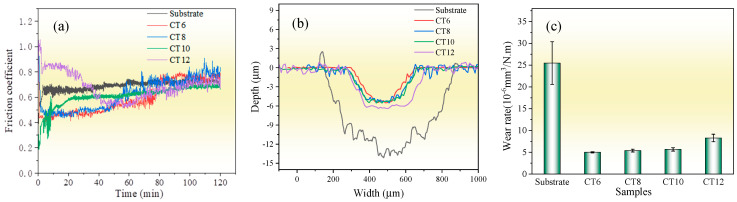
Wear properties of the substrate and the chromized layer (**a**) friction coefficient; (**b**) wear trace contour; (**c**) wear rate.

Figure 7 shows the friction and wear properties of the substrate and the chromized layer at room temperature. The average friction coefficients of the substrate, CT6, CT8, CT10 and CT12, are 0.69, 0.59, 0.62, 0.61 and 0.69, respectively. For CT6, CT8 and CT10, the average friction coefficient is lower than that of the substrate. However, with the increase in sliding time, their friction coefficient gradually increases and tends to be the same as the substrate at the end of the friction test. It is known that the main wear form of the GCr15 matrix is severe abrasive wear; thus, the friction coefficient is high, and the wear is serious [8]. After chromizing treatment, the high-hardness phase of chromium carbide is formed on the surface, and the resistance to abrasive wear is improved, resulting in a lower initial friction coefficient. However, with the increase in sliding time, the friction coefficient of the chromized layer gradually increases due to the continuous accumulation of hard abrasive particles at the friction interface. When the chromizing time is increased to 12 h (CT12 sample), coarse grains are formed on the surface of the chromized layer (see Figure 2), which can increase the surface roughness, resulting in a larger initial friction coefficient. As the coarse grains are gradually smoothed out with continued sliding, the friction coefficient decreased gradually. After approximately 50 min of wear, the variation trend of friction coefficient of the CT12 sample is similar to that of other chromized layers.

From the wear scar profile, the substrate displays a wear scar dimension with 726 μm in width and 14.29 μm in depth, whereas the chromized layer is markedly reduced in wear scar dimension with smaller width and depth. The wear depths of the chromized layer are lower than their thickness, indicating that the chromized layers have not been worn through. Figure 7c shows the wear rate, calculated based on the wear scar profile. The wear rate of the substrate is 25.47×10^−6^ mm^3^·N^−1^·m^−1^, which is about five times higher than that of the chromized layers. This suggests that the wear rate is reduced greatly by chromizing treatment. This improvement in wear resistance can be attributed to the high hardness of the chromized layer and excellent interface bonding strength between the substrate and coating. The wear rates of CT6, CT8 and CT10 samples exhibited minimal variations, while that of the sample CT12 has increased. This may be related to the larger friction coefficient of CT12 samples.

**Figure 8:**
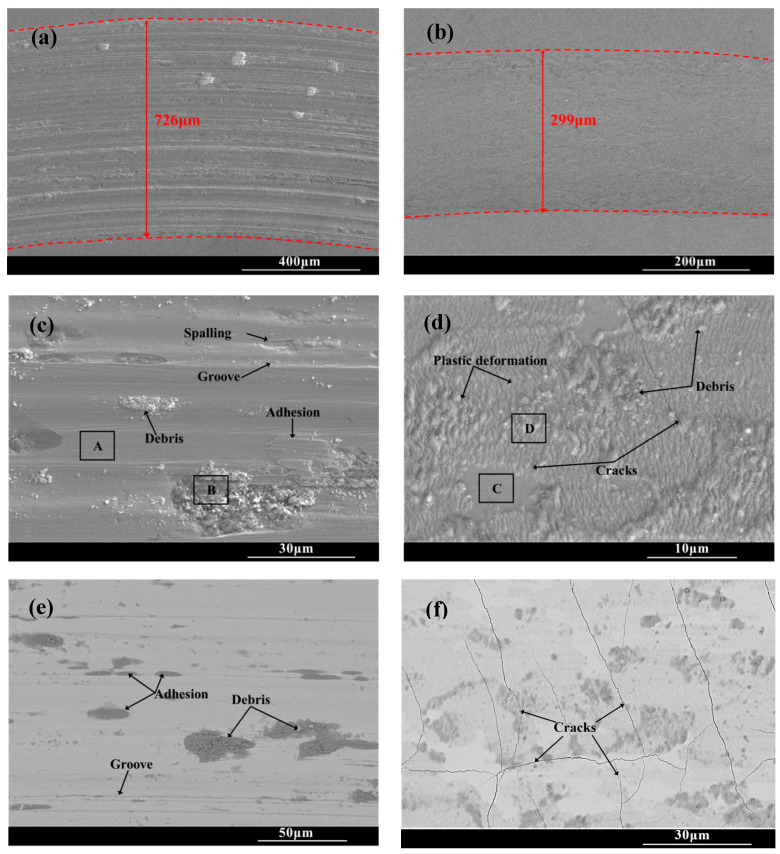
Wear trace morphology of the substrate and the chromized layer (**a**,**c**,**e**) substrate; (**b**,**d**,**f**) CT10.

Figure 8 shows the wear scar morphologies of the substrate and CT10 chromized layer after the friction and wear test. There are a large number of scratches, along with torn-wear phenomena within the wear scar of the substrate. Some wear debris adhere in the scar and are crushed to form abrasive particles. The EDS result shown in Table 3 reveals that oxidation is not obvious in the smooth Area A, but is severe in the abrasive particle’s Area B, which indicates that wear debris undergoes oxidation under repeated sliding of the load and forms hard oxide abrasive particles. The surface of steel substrate, mainly composed of Fe and a small amount of Cr, exhibits high oxygen affinity. During the repeated friction process, the interface temperature increases, causing these metals to combine with oxygen to form hard oxide particles. These hard particles embed in the wear interface, accelerating the abrasion of the soft substrate. Moreover, the affinity between the metal and the counter ball leads to adhesive wear, causing tearing and spalling of the material within the wear scar. Therefore, the wear mechanism of the GCr15 steel substrate is characterized by a combination of abrasive wear, adhesive wear and oxidative wear, collectively contributing to severe material wear.

The wear tracks of the chromized layer shown in Figure 8b,d,f exhibit relatively shallow profiles with minor superficial scratches, indicating that the superior wear is resistant to the chromized layer. Higher-magnification images (Figure 8d,f) reveal the presence of microcracks, plastic deformation zones and fine wear debris particles within the wear tracks. During the friction contact process, the material in the wear tracks is subjected to repeated shear stress and compressive stress, leading to plastic deformation as the material flows along the direction of the friction force. The thickness change in the deformation zone is inversely proportional to the hardness of the material. Generally, the material with large plastic deformation has lower hardness, while the material with higher hardness is more resistant to deformation. In the severe deformation zone, intense shear and contact stresses induce pronounced grain slip and flow, resulting in irregular deformation characteristics [46]. As observed in Figure 8d, it can be found that the plastic deformation zone within the wear scar of the chromized layer is shallow due to its high hardness. Under the cyclic contact stress and fatigue at the friction interface, micro-cracks nucleate at pores and grain boundary regions in the chromized layer. These micro-cracks gradually propagate under the repeated loading, eventually developing into macroscopic cracks within the wear tracks. As the crack density increases and propagates to form interlaced cracks, material spalling will be triggered.

It can be seen that although the substrate material undergoes extensive plastic deformation within the wear tracks due to its low hardness and high toughness, but no cracks are generated. However, the substrate is heavily ground due to its low hardness, resulting in poor wear resistance. In contrast, despite the presence of numerous cracks, the chromized layer demonstrates significantly enhanced wear resistance owing to its superior hardness and strong metallurgical bonding with the substrate.

Additionally, compared with that of the GCr15 steel substrate, no adhesive wear phenomenon is found within the wear tracks of the chromized layer. The chromized layer is mainly composed of hard ceramic phases: (Cr,Fe)_23_C_6_ and (Cr,Fe)_7_C_3_. The ceramic phases exhibit high chemical stability and poor affinity with the counter grinding ball. Therefore, the phenomena of adhesive wear and oxidative wear are significantly suppressed (as shown in Table 5). Thus, the main wear mechanism of the chromized layer is abrasive wear.

## 3. Experimental Section

### 3.1. Materials and Process

GCr15 bearing steel (provided by Luoyang Bearing Research Institute CO., Ltd., Luoyang, China) with dimensions of Φ30 mm × 5 mm was used as the substrate material. The chemical composition of the GCr15 bearing steel is given in Table 6. The samples were polished to obtain a mirror finish and ultrasonically cleaned in acetone and alcohol, then dried before the samples were chromized.

The chromized layer was fabricated by a solid powder pack chromizing method. The chromizing powder consisted of 50 wt.% chromium powder, 40 wt.% Al_2_O_3_ powder, 7 wt.% LaO_2_ powder and 3 wt.% NH_4_Cl (chromium powder, Al_2_O_3_ powder and LaO_2_ powder were provided by Zhongchun New Materials (Beijing) Technology Co., Ltd. China, Beijing, China, and NH_4_Cl was provided by Guangzhou Chemical Reagent Factory, Guangzhou, China). Firstly, the cleaned samples were embedded in the mixture powder within a steel box. Then, the chromizing treatment was carried out using a KBF13Q atmosphere electrical resistance furnace (Nanjing Nanda Instrument Co., Ltd., Nanjing, China). Before heating, the furnace was pumped to a pressure below −0.1 MPa, followed by argon purging until the pressure reached 0 MPa (equivalent to atmospheric pressure). The vacuum-pumping and gas-filling process was repeated three times to ensure an inert environment. The chromizing parameters were as follows: the heating rate of 10 °C/min, the holding temperature of 840 °C, and the holding times of 6 h, 8 h, 10 h, and 12 h (designated as CT6, CT8, CT10, and CT12, respectively). After holding, the samples were furnace-cooled. Subsequently, quenching treatment was performed at 840 °C for 30 min, followed by oil quenching. Annealing was conducted at 160 °C for 2 h, and the samples were then furnace-cooled. Finally, the samples were ultrasonically degreased and cleaned in acetone and alcohol before drying.

### 3.2. Characterization

The surface and cross-sectional morphologies of the chromized layer were characterized using a NOVA NanoSEM430 field-emission scanning electron microscope (FE-SEM) (Thermo Fisher Scientific, Waltham, Massachusetts, USA), while the elemental composition was analyzed with energy-dispersive spectroscopy (EDS). For cross-sectional analysis, samples were first sectioned using wire electrical discharge machining, followed by resin embedding, polishing to a mirror finish, and etching with a 4% nitric acid–alcohol solution. Phase structure analysis was carried out using a Philips X’Pert Pro MPD X-ray diffractometer (XRD) (Philips Analytical) with Cu Kα radiation. Both conventional Bragg–Brentano geometry and grazing-incidence XRD (GIXRD) at an incident angle of 2° were employed. Surface microhardness was tested using an MH-5D microhardness tester (Hengyi Precision Instrument Co., Ltd, Shanghai, China) under a load of 50 g and holding for 15 s. The average value was obtained by repeating 5 times. The adhesion strength was evaluated by a Rockwell indentation tester (Laizhou Huayin Test Instrument Co., Ltd, Shandong, China), using a 150 kg load. Three indentations were performed for each sample and then SEM was used to analyze the indent morphology. Friction and wear properties of both the substrate and chromized layer were investigated using a UMT-tribolab tribometer (Bruker, Billerica, MA, USA) at room temperature. The tribological experimental test conditions were presented in Table 7. The experiment was repeated three times. Wear track profiles were measured using a Dektak XT 3D profilometer (Bruker, Billerica, MA, USA), and the wear rate was calculated according to Equation (13):

(13) $$W=\frac{V}{LF_{N}}$$

where *V* is the wear volume (mm^3^), *L* is the sliding distance (m) and *F_N_* is the normal load (N).

## 4. Conclusions

The packing chromizing method was used to fabricate a chromized layer on the surface of GCr15 bearing steel. The microstructure, hardness, adhesion strength and tribological properties of the chromized layer were investigated.

The chromized layer exhibits a relatively smooth topography, with scattered protruding rhombic grains embedded on the surface. There are a certain number of fine pores existing on the surface and at the interface of the chromized layer/steel substrate. The content of the Cr element in the chromized layer gradually decreases from the surface to the interior, while the content of Fe element gradually increases, forming a good gradient structure. The content of C is high throughout the chromized layer. The chromized layer is mainly composed of (Cr,Fe)_23_C_6_ and (Cr,Fe)_7_C_3_ ceramic hard phase. With the increase in chromizing time, the microstructure of the chromized layer does not change obviously, except for the increase in thickness.

With the increase in chromizing time, the hardness of the chromized layer increases, ranging from 1360.6 HV to 1588.2 HV, which is more than threefold higher compared to that of the substrate material. A large number of radial cracks and circumferential cracks exist in and around the Rockwell indentation. As the chromizing time increases, the brittleness of the chromized layer increases and the spalling phenomenon of the chromized layer around the edge of the indentation is aggravated.

The wear mechanism of the GCr15 steel substrate is characterized by a combination of abrasive wear, adhesive wear and oxidative wear, collectively leading to severe material wear. In contrast, the wear behavior of the chromized layer is only slight abrasive wear, despite the occurrence of microcracks during the friction process. The average friction coefficient of the chromized layer has been reduced, and the wear rate is about one fifth of that of the substrate. This significant improvement in the wear resistance of the chromized layer is primarily attributed to its high hardness and strong metallurgical bonding with the substrate.

## Figures and Tables

**Table 1:** The reactions occur during the chromizing process.

Formulas	Gibbs Free Energy	Number
NH_4_Cl(s)→NH_3_ + HCl(g)		(1)
2NH_3_→N_2_ + 3H_2_(g)		(2)
2HCl(g) + Cr(s)→CrCl_2_(g) + H_2_(g)		(3)
CrCl_2_(g) + Fe→FeCl_2_ + [Cr] (Active Cr atoms)		(4)
CrCl_2_(g) + H_2_→2HCl(g) + [Cr]		(5)
CrCl_2_→Cl_2_ + [Cr]		(6)
2Cr + N_2_→2CrN		(7)
4Cr + N_2_→2Cr_2_N		(8)
23Cr + 6C→Cr_23_C_6_	ΔG_0_ (KJ mol^−1^) = −309.616 − 0.0774 T (298 K < T < 1773 K)	(9)
7Cr + 3C→Cr_7_C_3_	ΔG_0_ (KJ mol^−1^) = −174.401 − 0.0259 T (298 K < T < 2171 K)	(10)
3Cr + 2C→Cr_3_C_2_	ΔG_0_ (KJ mol^−1^) = −79.078 − 0.01766 T (298 K < T < 2130 K)	(11)

**Table 2:** Grain size of carbide under different chromizing time.

Sample	Cr_23_C_6_ (nm)	(Cr,Fe)_7_C_3_ (nm)
CT6	32.2	33.6
CT8	34.0	32.5
CT10	37.4	31.4
CT12	37.2	35.8

**Table 3:** EDS elements composition in a different position on the surface of chromized layers in Figure 2.

Position	Elements (at.%)		
	Cr	C	N
A	60.60	17.96	21.43
B	72.40	27.60	/
C	71.87	28.13	/

**Table 4:** EDS elements composition in different positions of the cross-section chromized layer in Figure 3.

Position	Elements (at.%)		
	Cr	C	Fe
A	49.03	49.67	1.30
B	50.33	47.46	2.21
C	24.29	49.59	26.11
D	10.20	43.56	46.23

**Table 5:** EDS element composition of the different positions of wear trace in Figure 8.

Position	Elements (at.%)			
	Cr	C	Fe	O
A	1.45	1.34	94.16	2.19
B	0.85	2.67	48.81	47.05
C	49.05	8.95	34.96	7.03
D	47.86	7.25	31.32	13.57

**Table 6:** Chemical composition of the GCr15 bearing steel.

Element	C	Cr	Mn	Si	Al	Cu	S	P	Fe
wt.%	1.0	1.4	0.3	0.2	0.1	0.04	0.03	0.01	balance

**Table 7:** Tribological experimental test conditions.

Conditions	Value
Normal force (N)	10
Sliding speed (m/s)	0.105
Sliding distance (m)	756
Pin-end Diameter, spherical (mm)	6
Pin-end material	Al_2_O_3_
Environment (Dry)	Atmospheric environment
Track diameter (mm)	10
Temperature (°C)	26
Humidity (RH)	52%

## Data Availability

Data is contained within the article.

## References

[1] Zhao Z., Xie X., Tang G., Padhiar M.A., Xiao J., Liang Z. (2023). Study on the effect of the strengthen grinding process surface coverage on the micro-morphology, micro-hardness, roughness, and residue stress of GCr15 bearing steels. J. Manuf. Process..

[2] Zhang H., Zheng Z., Qiao Z., Han D., Du A., Ma R., Fan Y., Zhao X., Cao X. (2025). Novel process used to accelerate spheroidizing annealing of GCr15 bearing steel by modulating initial structure. Mater. Today Commun..

[3] Zhang H., Jiao F., Lian X., Zhao Y., Niu Y., Tong J. (2025). Surface quality in ultrasonic-electrolytic internal grinding of GCr15 steel. Inte. J. Mech. Sci..

[4] Zhang W., Li W., Liu H., He T., Deng S., Tian H., Cao W. (2024). Microstructure effects on improving rolling contact fatigue by a double-quenching heat treatment process for GCr15 steel. Tribol. Int..

[5] Li Y., Ying S., Zhao Y., Li Z., Cao J., Wu X., Du P., Wu T., Yu C., Xie G. (2024). Study on fretting friction properties based on GCr15 alloy and liquid epoxy resin protective coatings on GCr15 alloy. Colloid. Surface. A.

[6] Mei S., Xiao Z., Chen Z., Hu Z., Zou X., Xu Q., Zheng Q. (2025). Prediction and optimization of Aluminum content in Ni-P-Al_2_O_3_ composite coatings on GCr15 Steel Based on PSO-BP Neural Network. Surf. Interf..

[7] Xie X., Guo Z., Liang Z., Xiao J., Zhao Z. (2023). Enhanced high-temperature wear resistance of GCr15 steel balls by generating a Ti + Nb diffusion layer via mechanical alloying and NH_3_.H_2_O treatment. Surf. Coat. Technol..

[8] Liu S., Li Y., Wang Y., Wei Y., Zhang L., Wang J. (2022). High wear resistance WC-Co reinforced GCr15 bearing steel composite prepared via selective laser melting (SLM). Int. J. Refract. Met. H..

[9] Wang W., Yuan W., Guo Q., Wang N., Chi B. (2024). Effect of picosecond laser surface texturing under Babbitt coating mask on friction and wear properties of GCr15 bearing steel surface. Eng. Fail. Anal..

[10] Xiao J., Xie X., Liu X., Li D., Zhou Y. (2024). Fabrication of gradient structure for enhancing wear resistance of GCr15 bearing steel friction shim. Wear.

[11] Li Y., Chen C., Fan Z., Jiang D., Shuai S., Tu T. (2024). Effects of cooling rate on isothermal spheroidizing annealing of hot-rolled GCr15 bearing steel. J. Mater. Res. Technol..

[12] Maskavizan A.J., Quintana J.P., Dalibón E.L., Márquez A.B., Brühl S.P., Farina S.B. (2024). Evaluation of wear and corrosion resistance in acidic and chloride solutions of Cathodic Arc PVD chromium nitride coatings on untreated and plasma nitrided AISI 4140 steel. Surf. Coat. Technol..

[13] Novák M., Novotný R., Valtr J., Dašek D., Cvrček L., Krejčí J., Vrtílková V., Macák J. (2024). The effect of chromium-based coatings on corrosion behavior of alloy Zr1Nb in 70ppm Li^+^ water environment. J. Nucl. Mater..

[14] Bai C.Y., Wen T.M., Hou K.H., Ger M.D. (2010). The bipolar plate of AISI 1045 steel with chromized coatings prepared by low-temperature pack cementation for proton exchange membrane fuel cell. J. Power Sources.

[15] Jathar S., Honnali S.K., Farhadizadeh A., Febvier A.l., Odén M., Eklund P. (2024). Influence of nitrogen and niobium incorporation in bcc-chromium coatings on microstructure and mechanical properties. Surf. Coat. Technol..

[16] Iliescu I., Gazal Y., Michau A., Addou F., Duguet T., Monsifrot E., Schuster F., Maury F. (2021). Low temperature Direct Liquid Injection MOCVD of amorphous CrCx coatings in large-scale reactors: An original route to nanostructured multilayer coatings. Surf. Coat. Technol..

[17] Roshan K., Jayakrishnan R., Thirumalai K.S. (2024). Corrosion measurement of thermally sprayed carbide coatings on stainless steel pipes. Results Surf. Interfaces.

[18] Beck K., Ulrich A.S., Thor N., Oskay C., Galetz M.C. (2024). Chromium diffusion coatings for improving the oxidation behavior of refractory metals at intermediate temperatures. Int. J. Refract. Met. H. Mat..

[19] Ye J.L., Ye F. (2011). Effects of strong carbide-forming elements on low temperature salt-bath chromizing. Adv. Mater. Res..

[20] Ganji O., Sajjadi S.A., Yang Z.G., Mirjalili M. (2022). Tribological properties of duplex coatings of chromium-vanadium carbide produced by thermo-reactive diffusion (TRD). Ceram. Int..

[21] Najari M.R., Sajjadi S.A., Ganji O. (2022). Microstructural evolution and wear properties of chromium carbide coating formed by thermo-reactive diffusion (TRD) process on a cold-work tool steel. Results Surf. Interfaces.

[22] Liu T., Wang C., Meng Q., Song Q., Xue B., Zhang Y., Cheng H., Wang Y. (2024). Effect of steel matrix with different C content on the growth mechanism, microstructure and properties of the chromized layer. Surf. Coat. Technol..

[23] Wei C.B., Lin S.S., Dai M.J., Shi Q., Su Y., Tang P. (2021). Structure and Performance Stability of Chromized Coatings by Pack Cementation. Rare Met. Mat. Eng..

[24] Dong Z., Zhou T., Liu J., Zhang X., Shen B., Hu W., Liu L. (2019). Effects of pack chromizing on the microstructure and anticorrosion properties of 316L stainless steel. Surf. Coat. Technol..

[25] Dong Z., Zhou T., Liu J., Zhang X., Shen B., Hu W., Liu L. (2019). Performance of surface chromizing layer on 316L stainless steel for proton exchange membrane fuel cell bipolar plates. Int. J. Hydrog. Energ..

[26] Lee S.B., Cho K.H., Lee W.G., Jang H. (2009). Improved corrosion resistance and interfacial contact resistance of 316L stainless-steel for proton exchange membrane fuel cell bipolar plates by chromizing surface treatment. J. Power Sources.

[27] Liu S., Yang J., Liang X., Sun Y., Zhao X., Cai Z. (2022). Investigation of the preparation, corrosion inhibition, and wear resistance of the chromized layer on the surfaces of T9 and SPCC steels. Materials.

[28] Lee J.W., Duh J.G. (2004). Evaluation of microstructures and mechanical properties of chromized steels with different carbon contents. Surf. Coat. Technol..

[29] Tan C., Zong X., Zhou W., Cao H., Wang J.J., Wang C., Peng J., Li Y., Li H., Wang J.S. (2022). Insights into the microstructure characteristics, mechanical properties and tribological behaviour of gas-phase chromized coating on GCr15 bearing steel. Surf. Coat. Technol..

[30] Wang H.F., Wang C.M., Liu T.T., Song Q., Xue Y.L., Ma Q.C., Cui H.Z. (2023). Growth Mechanism and Pore Formation of Solid Chromizing Layer of GCr15 Pin. Rare Met. Mat. Eng..

[31] Zong X., Jiang W., Fan Z. (2019). Evaluation of chromium carbide coatings on AISI 52100 steel obtained by thermo-reactive diffusion technique. Mater. Sci..

[32] Vidakis N., Antoniadis A., Bilalis N. (2003). The VDI 3198 indentation rest evaluation of a reliable qualitative control for layered compounds. J. Mater. Process. Technol..

[33] Wu C., Hong Y., Chen W., Chen J., Yuan M., Liao X. (2016). A double strengthened surface layer fabricated by nitro-chromizing on carbon steel. Surf. Coat. Technol..

[34] Guo S., Liu L., He F., Wang S. (2024). Preparation of Cr-N coatings on 316H stainless steel via pack chromizing and gas nitriding, and their resistance to liquid metal corrosion in early stages. Surf. Coat. Technol..

[35] Li L.B., Li Z.X., Liu L.T., He F. (2021). Effect of reaction temperature and time on microstructure of chromizing layer on austenitic stainless steel matrix. Rare Met. Mat. Eng..

[36] Hu J., Zhang Y., Yang X., Li H., Xu H., Ma C., Dong Q., Guo N., Yao Z. (2018). Effect of pack-chromizing temperature on microstructure and performance of AISI 5140 steel with Cr-coatings. Surf. Coat. Technol..

[37] He Y., Huang G., Liu Y., Tang Y., Fu X., Bao H., Ge M., Huang H., Zhang R., Liu H. (2024). A simple and feasible method for preparing chromium carbide coating on graphite through binary NaCl-KCl molten salt. Mater. Today Commun..

[38] Iorga S., Cojocaru M., Chivu A., Ciuca S., Burdusel M., Badica P., Leuvrey C., Schmerber G., Ulhaq-bouillet C., Colis S. (2014). Influence of the Carbo-Chromization Process on the Microstructural, Hardness, and Corrosion Properties of 316L Sintered Stainless Steel. Metall. Mater. Trans. A.

[39] Arzamasov B.N., Mel’nikov R.A. (1994). Investigation of the pore-formation process during the chromizing of steel 40 Kh by the circulation method. Met. Sci. Heat Treat..

[40] Lin N., Guo J., Xie F., Zou J., Tian W., Yao X., Zhang H., Tang B. (2014). Comparison of surface fractal dimensions of chromizing coating and P110 steel for corrosion resistance estimation. Appl. Surf. Sci..

[41] Cao H., Luo C.P., Liu J., Zou G. (2007). Phase transformations in low-temperature chromized 0.45 wt.% C plain carbon steel. Surf. Coat. Technol..

[42] Meng T.X., Guo Q., Xi W., Ding W.Q., Liu X.Z., Lin N.M., Yu S.W., Liu X.P. (2018). Effect of surface etching on the oxidation behavior of plasma chromizing-treated AISI440B stainless steel. Appl. Surf. Sci..

[43] Burnett P.J., Rickerby D.S. (1987). The mechanical properties of wear-resistant coatings I: Modelling of hardness behaviour. Thin Solid Film..

[44] Fu K., Yin Y., Chang L., Shou D., Zheng B., Ye L. (2013). Analysis on multiple ring-like cracks in thin amorphous carbon film on soft substrate under nanoindentation. J. Phys. D Appl. Phys..

[45] Liu J.N., Xing Z.G., Wang H.D., Cui X.F., Jin G., Xu B.S. (2019). Microstructure and fatigue damage mechanism of Fe-Co-Ni-Al-Ti-Zr high-entropy alloy film by nanoscale dynamic mechanical analysis. Vacuum.

[46] Borodin E., Bushuev O., Bratov V., Jivkov A.P. (2024). Discrete model for discontinuous dynamic recrystallisation applied to grain structure evolution inside adiabatic shear bands. J. Mater. Res. Technol..

